# Targeting Innate Immunity to Combat Cutaneous Stress: The Vitiligo Perspective

**DOI:** 10.3389/fimmu.2021.613056

**Published:** 2021-04-14

**Authors:** Katia Boniface, Thierry Passeron, Julien Seneschal, Meri K. Tulic

**Affiliations:** ^1^ Univ. Bordeaux, INSERM, BMGIC, U1035, Immuno-dermatology Team, Bordeaux, France; ^2^ INSERM U1065, Centre Méditerranéen de Médecine Moléculaire (C3M), Côte d’Azur University, Nice, France; ^3^ Côte d’Azur University, Department of Dermatology, CHU Nice, Nice, France; ^4^ Department of Dermatology and Pediatric Dermatology, National Reference Center for Rare Skin Disorders, Hôpital Saint-André, Bordeaux, France

**Keywords:** innate immunity, vitiligo, PAMPs, DAMPs, ILC, DC, melanocytes

## Abstract

Multiple factors are involved in the process leading to melanocyte loss in vitiligo including environmental triggers, genetic polymorphisms, metabolic alterations, and autoimmunity. This review aims to highlight current knowledge on how danger signals released by stressed epidermal cells in a predisposed patient can trigger the innate immune system and initiate a cascade of events leading to an autoreactive immune response, ultimately contributing to melanocyte disappearance in vitiligo. We will explore the genetic data available, the specific role of damage-associated-molecular patterns, and pattern-recognition receptors, as well as the cellular players involved in the innate immune response. Finally, the relevance of therapeutic strategies targeting this pathway to improve this inflammatory and autoimmune condition is also discussed.

## Introduction

Clinical, translational, and fundamental research studies performed over the last decade have tremendously improved our understanding of vitiligo physiopathology and new therapeutic perspectives are emerging for this disease which suffers from the lack of effective treatments. Vitiligo is a puzzling disease combining multiple intertwined components including environmental triggers, genetic predisposition, increased oxidative stress, and abnormal immune and inflammatory response (1, 2). Vitiligo is defined by the loss of epidermal melanocytes, nonetheless several cell subsets of immune and non-immune cells are involved to induce and/or contribute to their disappearance. Vitiligo skin is consistently associated with infiltration of T cells with a Th1/Tc1 skewed immune profile which target melanocytes (3, 4). Besides the role of the adaptive immune response, increasing data highlight a major role of innate immune cell subsets and their immune-related pathways that could spark the induction of the disease in the “normal-appearing” skin. Therefore, this short review is focusing on the innate side of the disease, discussing how genetic and transcriptomic data revealed the importance of innate immunity in vitiligo, as well as the interplay between epidermal cells (keratinocytes and melanocytes) and innate immune cells to contribute to the initiation and/or progression of the disease through the release of danger signals, cytokines, and chemokines, leading to activation of the adaptive immune response and ultimately the loss of melanocyte. This better understanding now offers novel insight into the development of targeted therapies that could prevent the induction as well as the recurrence of the disease.

## Genetic and Transcriptome Data

Genome wide association studies (GWAS) have identified over 50 susceptibility loci involved in melanogenesis and immunity in vitiligo patients (5). On the other hand, a delay in vitiligo age-of-onset over the past 30 years emphasizes the key role of environmental factors in triggering vitiligo in genetically predisposed individuals (6, 7). These GWAS studies not only demonstrated the implication of genes involved in melanogenesis and adaptive immunity but also revealed allelic variations in key genes involved in the innate immune responses, such as IFIH1, NLRP1, or TICAM1 (7–9).

Transcriptional analysis comparing gene expression profiles of skin from vitiligo patients with normal skin of healthy volunteers also emphasized the role of innate immunity (10, 11). Thus, natural killer (NK) cell activation markers, such as NKG2D, KLRC2, and KLRC4, ligands for NK receptor (CLEC2B), as well as markers of oxidative stress (CANP and POSTN) and innate immunity (DEFB103A) were shown to be increased in vitiligo skin (10). In our study, we also found a significant increase in NK receptors, including NKTR and KLRC1, as well as trends for increased EOMES (master regulator of NK cells), CCL20, and NK-related cytokines (TNFα and IL-15) (11). Interestingly, activation of these innate immunity markers was found in the non-lesional skin of vitiligo patients, suggesting that the activation of the innate immunity may be present throughout the entire skin surface of patients (10, 11).

Taken together, these data illustrate that vitiligo patients have genetic predisposition affecting their innate immune response in their apparent non-affected skin. Such findings may be indicative of a subclinical activation of innate immunity, loss of protective mechanisms to stress (such as defective unfolded protein response in target cells following endoplasmic reticulum stress), and/or increased sensitivity to endogenous or external stress, such as several damage-associated-molecular patterns (DAMPs) or pathogen-associated-molecular patterns (PAMPs) (12).

## Activation Of Innate Immune Cells by Danger Signals

### DAMPs

Several DAMPs have been detected in perilesional skin of vitiligo patients. Previous studies have shown that the chromatin-associated nuclear protein High-mobility group-box-1 (HMGB1) could be released by melanocytes under oxidative stress and could directly impact melanocyte survival (13–15). Additionally, HMGB1 could bind free DNA and HMGB1-DNA complexes and induce maturation of vitiligo patients’ dendritic cells (DC), as well as the production of cytokines and chemokines by keratinocytes (16). Another candidate for sensing the immune system in vitiligo is calreticulin (CRT). In response to stress, CRT can localize at the surface of immune cells, affecting their antigen presentation, complement activation, and clearance of apoptotic cells. Moreover, CRT can translocate to the melanocyte surface when these cells undergo H_2_O_2_-mediated oxidative stress, increasing melanocyte immunogenicity. CRT may also enhance the immunogenic potential of melanocytes through their induction of pro-inflammatory cytokine production, such as IL-6 and TNFα (17).

Heat shock proteins (HSP) are likely important candidates bridging stress to the skin with the innate immune response. Indeed, inducible HSP70 (HSP70i) released in the context of cellular stress, notably by epidermal cells (including keratinocytes and melanocytes) has been shown to accelerate the progression of the disease in a preclinical model (18–20). Likewise, modified HSP70i prevented or reversed vitiligo in a mouse and Sinclair Swine models of the disease (21, 22). In vitiligo patients, the expression of HSP70 in the skin correlated with disease activity and was lower in patients with stable disease (23). As discussed below, HSP70i could interact with several cell subsets, leading to their activation.

### Pattern Recognition Receptors

PAMPs are critical in initiation of the innate immune response through activation of pattern recognition receptors (PRRs). Implication of PRRs in vitiligo has been demonstrated in several GWAS, in particular genes encoding TLRs and their signaling pathway (24, 25). In addition, polymorphisms in NLRs have been described in patients with non-segmental vitiligo. Upregulated NLRP3 expression has been detected in perilesional keratinocytes in vitiligo skin and associated with higher cutaneous IL-1β expression and increased severity of the disease (26, 27).

Viral components are likely involved in vitiligo pathogenesis, as they can trigger activation of the immune system, however whether viruses can activate the innate immune response in the context of vitiligo is poorly described. Viruses possess several structurally diverse PAMPs, including surface glycoproteins, DNA, and RNA species (28). Virus infection could thus activate the innate response and potentially trigger a vitiligo flare. There is some evidence that viral infections in a genetically predisposed host may induce excessive ROS production by recruited lymphocytes leading to destruction of epidermal melanocytes (29). Furthermore, IFIH1, encoding intracellular virus sensor MDA5, has been identified as a vitiligo susceptibility gene capable of inducing secretion of CXCL10 and CXCL16 from keratinocytes and inducing infiltration of CD8^+^ T cells in vitiligo (30).

Bacteria are among the top producers of PAMPs and could directly trigger PRRs activation and therefore participate in activation of the innate immune response in vitiligo, however their direct role in triggering vitiligo has yet to be proven. While gut dysbiosis has been reported in several auto-immune disorders, there exists only one study suggesting skin dysbiosis in lesional zone of vitiligo patients compared to their non-lesional skin; however in that study there was no comparison to skin microbiota from healthy skin (31). The second study in mouse model of vitiligo treated with antibiotics has shown that depletion of certain bacterial strains in the gut induces skin depigmentation, suggesting possible gut-skin axis in the disease (32). We have recently demonstrated gut and skin dysbiosis in vitiligo compared to healthy controls; the most striking differences were seen in the deeper regions of vitiligo skin (33). Importantly, these changes were associated with mitochondrial damage and loss of protective bacteria at the same site with elevated systemic innate immunity in vitiligo patients.

## Role Of Innate Immune Cells in Vitiligo

As suggested above, a large number of innate immunity genes that confer risk for vitiligo have been identified in genetic studies. Collectively, these papers undeniably support innate immunity pathways as critical in the development of the disease, which was further confirmed at the transcriptional level, with increased expression of innate immune related genes both in non-lesional and lesional skin of vitiligo patients (10, 11). If there is activation of innate immune pathways, naturally we would expect to see influx or activation of resident innate immune cells in the skin of vitiligo patients; however, the data in this area of research is only now starting to emerge. Although it has been known for a while that there is infiltration of macrophages, inflammatory DCs, dermal DCs, Langerhans cells, and NK cells to the leading edge or the lesional sites (34–37), their roles in vitiligo have not been thoroughly explored. The contribution of inflammatory DCs (CD11c^+^ CD11b^+^) has been demonstrated in DAMP-induced animal model of vitiligo driven by HSP70 (21), however their role in human disease remains to be proven. Studies dating over 10 years ago have demonstrated a positive correlation between levels of macrophage migration inhibitory factor (MIF) in the blood of vitiligo patients and their disease duration suggesting MIF may be a useful serum biomarker of vitiligo activity (38–40), however direct contribution of macrophages to the disease in the skin is unknown.

### Plasmacytoid Dendritic Cells

Plasmacytoid dendritic cells (pDCs) certainly represent an important player in the initiation of the inflammatory response and the type I/II IFN signature in vitiligo skin. pDCs have been involved in various chronic inflammatory dermatoses, including cutaneous lupus erythematosus and psoriasis, mainly through their propensity to release high levels of IFNα (41). We showed that perilesional skin of vitiligo patients in the active phase of the disease harbors infiltrates of pDCs, associated with a local IFN response (36). Activation of this cell subset is likely mediated by the release of DAMPs from epidermal cells, as shown with HSP70i, potentiating IFNα secretion by pDCs and subsequent production CXCL9 and CXCL10 chemokines by epidermal cells, leading to the Th1 adaptive immune response establishment characteristic of vitiligo skin (42).

Furthermore, the other question which has been puzzling researchers was the initial source of the signature vitiligo cytokine IFNγ. We know that IFNγ is critical for the progression of vitiligo through 1) its induction of CXCL9 and CXCL10 chemokines and thereby recruitment of CD8^+^ T cells expressing CXCR3, which are without doubt responsible for the loss of melanocytes and 2) its direct effect together with TNFα on melanocyte, through induction of melanocyte detachment from the basal layer of the epidermis (43). We recently highlighted that type 1 innate lymphoid cells (ILC1) are also poised to release IFNγ (37).

### NK Cells

NK cells are described as a bridge between innate and adaptive immune system. They are characterized by their early and potent production of IFNγ. As discussed above, the transcriptional data supporting role of innate immunity in vitiligo is primarily based on differential gene expression associated with NK cell function, activity, and cytotoxicity (10, 11). It has been known for almost 30 years that there is an increase in circulating NK cells in the blood of vitiligo patients with abnormalities in their expression of inhibitory receptor CD158a and their activity (44–47), yet their role in vitiligo skin remained unexplored until recently. We have now confirmed the increased number of cytotoxic NK cells in not only the blood but also in the skin of vitiligo patients compared to healthy controls, predominantly in non-lesional skin (37). Furthermore, vitiligo NK cells are much more sensitive to stress, produce much larger amounts of IFNγ following stress, and are directly implicated in initiation of long-term adaptive immunity against melanocytes (37).

### Innate Lymphoid Cells

Innate lymphoid cells (ILCs) are the innate counterparts of T cells. In response to IL-12, IL-15, and IL-18, they secrete IFNγ; a signature vitiligo cytokine. We have recently demonstrated increased presence of ILC1 (but not ILC2 or ILC3) in vitiligo blood and skin and these cells to be the initial source of IFNγ, which is involved in early melanocyte apoptosis and subsequent T-cell mediated destruction of melanocytes (37).

### Melanocytes

It has been known for a long time that melanocytes from vitiligo patients are intrinsically abnormal and are more sensitive to external stress (48, 49), however this defect alone doesn’t explain the disease pathology as stressed melanocytes remain viable. As shown in a chicken model of spontaneous vitiligo, innate immunity is an important link between melanocyte stress and long-term adaptive immunity (50). Melanocytes have also been proposed as immunocompetent cells being able to process and present antigen, upregulate their own co-stimulatory markers and directly stimulate cytotoxic T lymphocytes following IFNγ stimulation (51, 52). We have recently shown that human melanocytes express chemokine receptor of the B-isoform (CXCR3B), whose expression is upregulated in vitiligo melanocytes compared to healthy melanocytes and this receptor to play a critical role in anti-melanocyte immunity in vitiligo (37).

Together, recent literature highlighted that innate immune pDCs, NK cells and ILC1 are capable of directly responding to stressed melanocytes and are critical in initiation of the disease, making these cells ideal primary target for therapeutic intervention.

## Therapeutic Perspectives

Vitiligo is a chronic inflammatory skin disorder and future therapeutic strategies might consider targeting the innate immunity side of the disease to halt initiation and/or progression of the disease, but such approach could also be envisioned as a maintenance therapy to prevent relapse.

Topical or systemic immunosuppressive drugs that are actually used for treating vitiligo such as corticosteroids, methotrexate or calcineurin inhibitors, have some potential impact on the innate immune response (53, 54). However, these agents have a broad impact on innate and adaptive immunity. Future approaches targeting more specifically the pathways involved in vitiligo could provide better responses with safer profile.

The elicitation of DAMPs depends on endoplasmic reticulum stress and oxidative stress. Many studies have described the role of the oxidative stress in vitiligo and how it can trigger the immune response (55–58). However, the efficacy of antioxidants in treating vitiligo is still a matter of debate as it relies on inconclusive studies or studies with contradictory results (59). This discrepancy between robust fundamental evidences and questionable clinical data could be explained by the differences in the type of antioxidant therapies used. More effective antioxidants with better bioavailability could effectively reduce the oxidative stress in the skin and provide a useful approach in treating vitiligo. There is increasing evidence showing mitochondrial alterations with increased production of ROS in vitiligo skin (60, 61). Compounds protecting specifically against this kind of mitochondrial damages could be of great interest in treating or preventing vitiligo relapses. Inhibition of DAMPs released by epidermal cells could also represent an interesting approach to prevent activation of innate cells. Indeed, mutant HSP70i have been shown to prevent auto-immune depigmentation or induce repigmentation both in mouse and Sinclair swine models (21, 22).

As detailed above, bacteria are major producers of PAMPs and alteration of skin and gut microbiome could participate in activation of the innate immune response in vitiligo (31–33). Modulating the skin or gut microbiome appears as an appealing approach. Recent data conducted in atopic dermatitis skin, demonstrated that topical formulation containing specific strains of probiotics could improve skin lesions (62). Additional studies are urgently needed, especially those in vitiligo, but modulation of microbiome, using prebiotics, probiotics, postbiotics, or fecal microbiota transplantation, could be an alternative approach for secondary prevention in vitiligo.

The development of antibodies targeting specifically the B isoform of CXCR3, could prevent the initial apoptosis of melanocytes and thus could be an effective preventive approach (37). Another strategy could rely on direct action on innate cells themselves. BDCA2 is a C-type lectin specifically expressed on pDCs, whose engagement inhibits the release of IFNα. Of interest, the use of a monoclonal antibody targeting BDCA2 showed improvement of skin lesions in systemic lupus erythematosus (63), however no preclinical studies have been performed so far to evaluate the efficacy of such strategy in vitiligo. In line with the important role of IFNα in disease pathogenesis, hydroxychoroquine, a TLR7 and TLR9 inhibitor downregulating IFNα production by pDCs, was shown to induce repigmentation of vitiligo lesions in a clinical case reports (64, 65). Whether inhibition of IFNα or its receptor could be an alternative strategy to block the type I IFN pathway in vitiligo has not yet been assessed.

NKG2D is one of the most frequent allelic variation found in vitiligo population. It also regulates both NK and T cell responses and thus, targets both innate and adaptive immune responses (66), and has been involved in vitiligo pathogenesis (67, 68). The use of anti-NKG2D antibodies could be a very promising approach for treating vitiligo.

In line with a therapy that would dampen both innate and adaptive immune response, IL-15 could represent another attractive strategy. Indeed, this cytokine is important for both T cells and NK cells maintenance and function. Recent studies highlighted the role of IL-15 on resident memory T cells in vitiligo pathogenesis and the interest to inhibit this cytokine or its receptor in vitiligo (68–70). Clinical phase II study is about to start, evaluating the efficacy of AMG714 for treatment of vitiligo (NCT04338581). Whether such targeting would also impact the innate response remains to be evaluated.

Targeting multiple cytokine pathways with JAK inhibitors is showing promising clinical outcome in vitiligo patients, as shown with the use of tofacitinib (blocking JAK1/3) or ruxolitinib (blocking JAK1/2) (71–73). Besides targeting IFNγ signaling, such therapies will also likely block IFNα impact on epidermal cells, as this cytokine signals through JAK1/TYK2. Interestingly, a phase 2 clinical trial evaluating the efficacy of systemic administration of a JAK1/TYK2 inhibitor is ongoing (NCT03715829) and will provide new insights into the physiopathology of vitiligo.

## Conclusion

**Figure 1:**
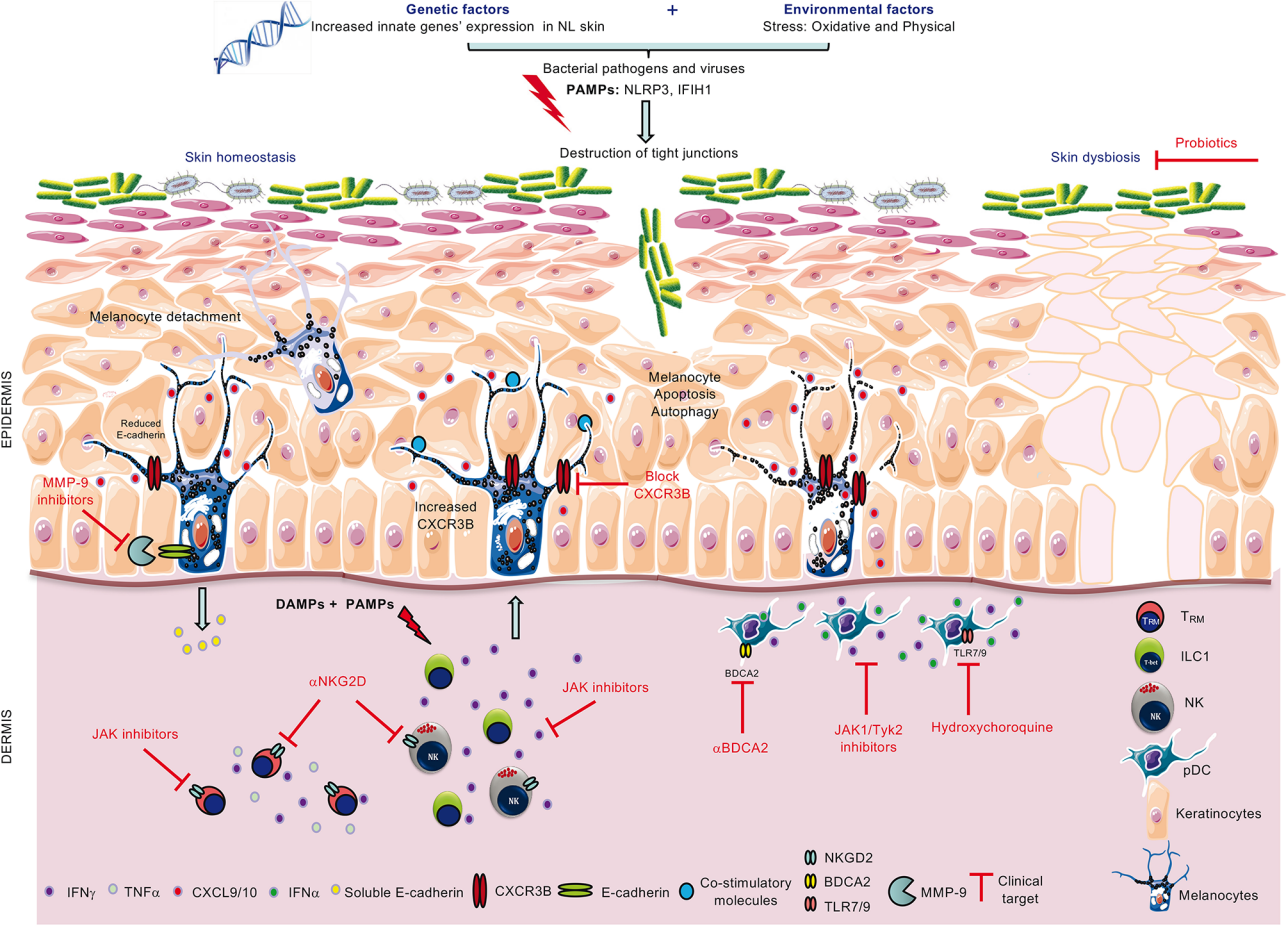
Role of innate immunity in loss of melanocytes and therapeutic strategies to combat the disease. Combined genetic and environmental (PAMPs) or endogenous (DAMPs) stress activates innate immune cells ILC1/NK and pDCs to increase their production of IFNγ and IFNα, respectively. In turn, IFNγ is involved in melanocyte detachment from the basal layer of the epidermis and upregulates melanocytic CXCR3B expression. In some melanocytes, CXCR3B activation induces melanocyte apoptosis which triggers increased expression of co-stimulatory molecules in other melanocytes and subsequent recruitment of cytotoxic T cells. Possible clinical therapeutic targets include the use of anti-CXCR3B (to block initial apoptosis), JAK inhibitors (to inhibit cytokine release from ILC1, pDCs and memory T cells, T_RM_), anti-NKG2D (to block activating NKG2D receptor on NK and T_RM_), anti-BDCA2 and hydroxychloroquine (to inhibit IFNα production from pDCs) and probiotics to restore skin homeostasis.

Innate immunity has long time been overlooked in autoimmune disorders, including in vitiligo. However, from genetic and transcriptome data to modulation of key innate cells in vitiligo skin and blood, there are now accumulating and strong evidences supporting the key role of the innate immunity in pathogenesis of vitiligo (**Figure 1**). Activated by the PAMPs and DAMPs, the innate immunity appears as the bridge between potential triggering factors of vitiligo flares such as stress, Koebner phenomenon or infections, and the secondary activation of the adaptative immune response. These data foster new therapeutic opportunities for vitiligo treatment but also for primary and secondary prevention. It will also be important to further characterize the role of the innate immune response in preventing repigmentation in patients with a stable disease.

## Author Contributions

KB, TP, JS, and MKT wrote the manuscript. All authors contributed to the article and approved the submitted version.

## Conflict of Interest

JS has been an advisor, speaker or investigator for Abbvie, Calypso Biotech, Lilly, Novartis, Pierre Fabre, Sanofi-Genzyme. KB has been an advisor, investigator for Calypso Biotech.

The remaining authors declare that the research was conducted in the absence of any commercial or financial relationships that could be construed as a potential conflict of interest.
